# Prediction of Thermal Modulated Comprehensive Two‐Dimensional Gas Chromatographic Separation Using a Modular, Graph‐Based Simulation Platform

**DOI:** 10.1002/jssc.70351

**Published:** 2026-01-21

**Authors:** Jan Leppert, Tillman Brehmer, Matthias Wüst

**Affiliations:** ^1^ Institute of Nutritional and Food Sciences Chair of Food Chemistry University of Bonn Bonn Germany

**Keywords:** comprehensive two‐dimensional gas chromatography, modular simulation, retention time prediction, thermal modulation

## Abstract

Comprehensive two‐dimensional gas chromatography (GC×GC) offers exceptional separation performance, but method development remains time‐consuming and sensitive to numerous system parameters. In this study, we present a modular simulation framework for GC×GC systems with thermal modulation, implemented in the open‐source Julia package GasChromatographySystems.jl. The simulation is based on a graph‐based abstraction of the GC system and models solute migration through column and modulator modules using previously established retention models. A simplified but effective model for thermal modulation enables the generation of realistic two‐dimensional retention times and peak widths. Simulation results were validated against experimental measurements from a GC×GC‐ToF‐MS system using different modulation periods and temperature programs. Systematic deviations between simulated and measured retention times could be explained and corrected by adjusting parameters such as the actual modulation period and modulator shift. The final model achieved root mean squared error (rmse) of below 15 s (less than 1%) for first‐dimension retention times and 55 ms (8%) for the second dimension. Peak width predictions were less accurate, with deviations of up to 3 s (40%) in the first dimension and up to 40 ms (60%) in the second. This modular and adaptable simulation framework provides a robust foundation for future applications in automated method development and system diagnostics in multidimensional gas chromatography.

## 1 Introduction

Multidimensional gas chromatography (MDGC) is a widely used separation technique across various fields of life science and analytical chemistry [1], especially in “omics” disciplines such as metabolomics and volatilomics [2, 3]. When coupled with mass spectrometry, comprehensive two‐dimensional GC (GC×GC) enables investigation and monitoring of complex biochemical processes in biological systems [4], supporting applications such as food authentication [5, 6] and medicinal diagnostics [7].

Due to the complexity of GC×GC systems, there is a growing need for efficient and systematic method development strategies. However, experimental method development remains time consuming and resource intensive. As a result, simulation‐based tools offer powerful means to accelerate this process in chromatography [8, 9, 10]. Digital tools and computational models not only reduce experimental workload but can also improve understanding of how instrumental parameters influence separation performance.

Although simulation tools for predicting retention times in one‐dimensional separations are available [11, 12, 13], suitable software solutions for multidimensional systems remain limited. However, promising approaches for GC×GC have emerged over the years [14, 15, 16, 17, 18, 19, 20, 21, 22]. Recently, Gaida et al. published a review about this topic [23]. These approaches typically simulate solute migration through GC columns using fixed time steps and terminate calculations once the solute reaches the column exit. While effective for certain use cases, these methods often lack flexibility in handling complex or highly customized setups.

With the growing use of machine learning and artificial intelligence (AI), data‐driven approaches are gaining relevance in predicting GC×GC and LC×LC separations [24, 25]. These methods offer promising alternatives but need large datasets available for training. The presented prediction model of GC×GC separation is based on physical and chemical models, which could be used in physical informed machine learning, increasing the accuracy and reducing the size of the needed training datasets.

Developments in instrument design have led to increasingly complex configurations combining MDGC, GC×GC, heart‐cut switching, cryogenic trapping, and olfactometry [26], or adding additional dimensions of separation, such as GC×GC×GC [27]. Emerging technologies like spatial thermal gradients [28, 29] expand the range of design possibilities of GC systems. Simulation can be a useful tool to understand separation dynamics and the interaction of additional parameters in these cases [30, 31].

A wide range of different modulation technologies is available, using two different approaches. The first technique is thermal modulation, in which the analytes are immobilized after eluting from the first‐dimension column by a reduced temperature, trapping the analytes by condensation or by adsorption on a stationary phase. The trapped analytes are then released rapidly by a rapid increase in temperature, resulting in a focused reinjection of the analytes onto the second dimension column. The second technique is flow modulation, which uses fast‐changing flows to direct portions of the eluate from the first‐dimension column onto the second‐dimension column. To trap the eluate from the first‐dimension sample loops are used. For further information about different modulators used in GC×GC, we refer to the review by Bahaghighat et al. [32].

This study presents a modular approach for numerically simulating the separation of volatile compounds in complex GC systems, building upon an open‐source programming toolbox [12, 33, 34]. A flow calculator based on this modular approach for arbitrary complex capillary networks was previously introduced [35]. In contrast to other GC×GC simulation concepts, the approach presented in this work uses a modular system architecture that abstracts the GC setup as a directed graph of interconnected modules (e.g., columns, modulators). Each module is simulated individually, and solute migration is modeled using ordinary differential equations (ODEs) solved with adaptive step‐size solvers (e.g., Runge–Kutta methods). This enables faster and more accurate calculation of retention times and peak widths. Moreover, the modular design inherently supports extensions to more complex configurations, such as GC×GC×GC systems, different modulator types, or systems with multiple detectors and switching devices, as well as different sample introduction techniques.

The performance of the simulation is demonstrated using thermally modulated GC×GC measurements, comparing predicted and experimental two‐dimensional retention times and peak widths across a range of measurement conditions. However, the resulting effect of flow modulation is similar to thermal modulation (periodic reinjection of analytes onto a second‐dimension column) and therefore the approach for the prediction of chromatograms using the presented modular system would also be similar.

## 2 Methods

### 2.1 GasChromatographySystems.jl

The open‐source software used for simulating GC separations in complex systems is published as a registered package for the Julia programming language. The source code and additional documentation are available on GitHub [36]. A list of symbols can be found in Table S1.

#### 2.1.1 Abstraction as Graph

A complex GC system, consisting of columns, transfer lines, and modulators, can be abstracted as a directed graph. In this representation, edges correspond to capillaries or modules, and vertices correspond to connection points (e.g., junctions, inlets, or outlets). Each vertex is associated with a local pressure, defining the inlet, respectively outlet, pressure of the adjected capillary segment.

In prior work [35], a general method was developed to compute pressure and flow distributions throughout such networks. Flow balance equations are automatically generated at each vertex based on mass conservation, resulting in a linear system in the squared pressures. Solving this system yields volumetric flow rates, pressures, and hold‐up times across the entire network.

This graph‐based approach enables automated, system‐wide analysis of complex GC configurations with multiple outlets, switching devices (e.g., Deans switches), or modulation elements. Accurate knowledge of local flow conditions is essential for subsequent chromatographic simulation, as retention times and dispersion depend sensitively on flow rate and pressure profiles. The flow calculator thus provides a consistent foundation for detailed modeling of GC separation processes.

#### 2.1.2 Modules of a System

In this presented work, two types of modules are used: a column module (CM) and a thermal modulator module (TM). Additional module types, e.g., a flow modulator module (FM) or injectors, could be added in the future. These modules represent capillaries of the GC system and form the edges in the graph abstraction of the system.

A CM is defined by its physical dimensions (length L, diameter d, film thickness $d_{f}$), the type of stationary phase, the temperature of the column (which can be a temperature program), the flow F, and additional options. The flow can be undefined if it should be calculated based on pressure values, see Table S2.

A TM shares similar parameters with a CM, but includes additional parameters: modulation period $t_{\mathrm{MP}}$, time shift $t_{\mathrm{shift}}$, durations of active cold jet $t_{\mathrm{cold}}$ and active hot jet $t_{\mathrm{hot}}$, relative temperature increase during the hot jet $T_{\mathrm{hot}}$, and the cold jet temperature $T_{\mathrm{cold}}$ (either as absolute value or as relative offset), see Table S3. The periodic temperature profile of the thermal modulator is approximated as a periodic rectangular function defined by these parameters, see Figure S1. The resulting temperature program of the TM is the sum of the rectangular function and the temperature program T, respectively the temperature during the cold jet is set to $T_{\mathrm{cold}}$ (depending on the selected option).

#### 2.1.3 Modular Simulation

Solute migration and peak broadening are simulated using a previously developed simulation tool GasChromatographySimulator.jl [12, 30, 37]. Each module in the system is defined by its properties, and the pressure p(x,t) is calculated across all points x and times t using the graph representation [35]. Possible flow paths are determined from inlet (source‐only) vertices to outlet (sink‐only) vertices, passing through a series of modules and connection points.

Solute migration proceeds module by module: for each module i, migration time $t_{R,i,s}$, and peak width $\tau_{R,i,s}$ for solute s are computed based on module parameters ($L_{i}$, $d_{i}$, $T_{i}\left(x,t\right)$, etc.) and the pressures of inlets and outlets ($p_{in,i}$, $p_{out,i}$), using the solutes entry time and peak width from the preceding module. The resulting migration time $t_{R,i,s}$ and peak width $\tau_{R,i,s}$ are then used as initial values for the following module i+1.

**FIGURE 1:**
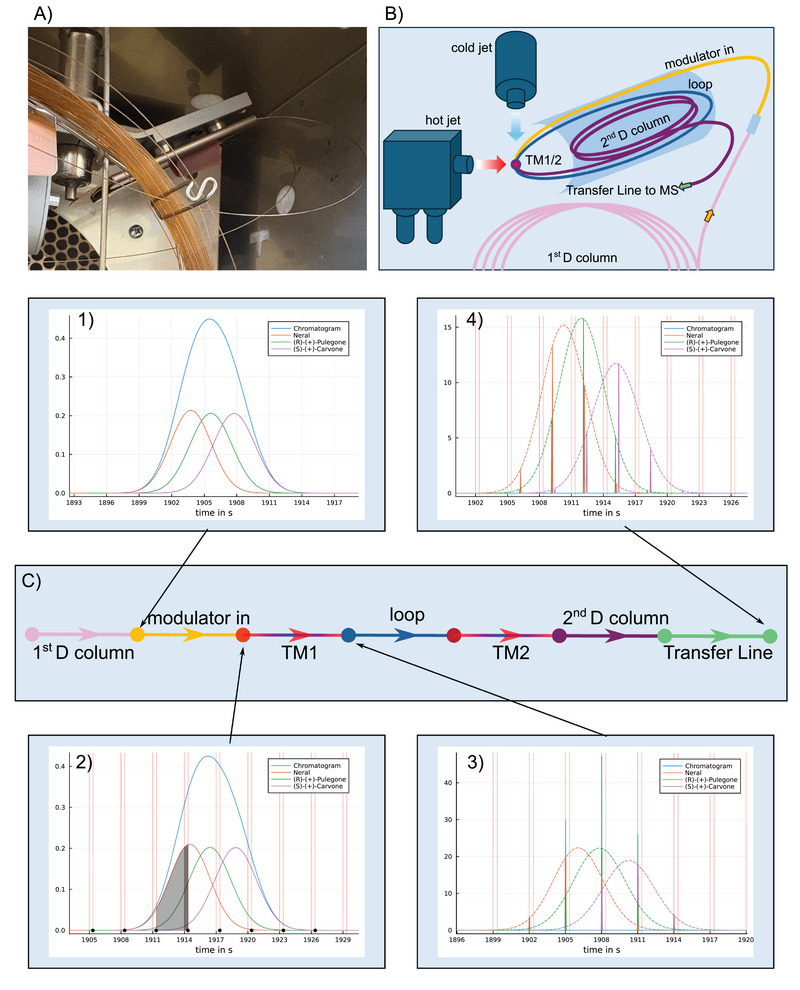
(A) Photo and (B) scheme of the used ZX 2 looped thermal modulator together with the corresponding graph representation of the GC×GC system, C). In the scheme and the graph corresponding segments are in the same color. (1) Output chromatogram of three overlapping solutes after the first‐dimension column. (2) Chromatogram after the initial segment of the second‐dimension column. In addition, the slicing of the solute peaks outlined by the area under the peak during a modulation period. Pink vertical lines indicate the begin/end of the hot jet. (3) Sliced and focused peaks after release with the next hot jet at the first modulator point “TM1.” (4) Final simulated raw chromatogram with separated peaks in the second dimension.

This principle is illustrated in Figure 1. For example, the result from the module “first D column” (Figure 1, subfigure 1) serves as input for the subsequent “modulator in” segment, a section of the second‐dimension column before the first modulation point. The simulation result of this segment is shown in Figure 1 (subfigure 2), which is the input data for the next module “TM1,” the first modulation point.

#### 2.1.4 Simulation in TM

Thermal modulators process the input peaks from the previous module into multiple slices. The modulator acts as a temporal sampling device, dividing continuous solute peaks into discrete slices based on the modulation period $t_{\mathrm{MP}}$. Each slice corresponds to the area under the Gaussian peak for one modulation cycle. Segments that elute during the hot jet (dark gray area in Figure 1, subfigure 2) are included in the slice approximation. In practice, this unfocused portion is not retained in the modulator during the hot phase but continues through it. Two‐stage modulators are assumed to recover and focus this portion.

The migration through the modulation module TM is also approximated. Each sliced segment is assumed to elute at the start of the next hot‐jet phase $t_{\mathrm{next}\;\mathrm{hot}}$. The retention time $t_{R}$ is calculated as:

(1) $$\;t_{R}=t_{\mathrm{next}\;\mathrm{hot}}+t_{\mathrm{migration}}$$

with migration time $t_{\mathrm{migration}}$ as the time required for the solute to pass through the modulator:

(2) $$\;t_{\mathrm{migration}}=\frac{L}{u_{R}}=L\frac{1+k}{u_{M}}$$

where $u_{M}$ is the mobile phase velocity, k is the retention factor at the modulator temperature, computed by adding the hot‐jet offset to the current oven temperature. L is the length of the modulation segment

The peak width $\tau_{R}$ is approximated as equal to the migration time:

(3) $$\;\tau_{R}=t_{\mathrm{migration}}\;$$

This simplified model avoids full numerical simulation while providing accurate approximations of the retention and dispersion behavior of modulated slices, enabling efficient modeling of peak focusing and release in thermal modulators. Using the full numerical simulation produced comparable retention times and peak widths, but required significantly higher computation time, by several orders of magnitude.

#### 2.1.5 Determination of Retention Times and Peak Widths of the Simulated 2D Peaks

The final result of the simulation is the peak list, collecting the raw retention times, peak widths, and areas of the multiple peaks of the separated substances. A chromatogram, similar to the raw chromatogram of a GC×GC measurement, can be constructed from this information by folding up the raw retention times.

The first‐dimension retention time of each slice of the same substance is the retention time corresponding to the number of modulations at elution multiplied by $t_{\mathrm{MP}}$. Projecting the heights of the modulated peaks with their corresponding modulation times onto the first‐dimension axis results in a distribution representing the profile of the substance peak in the first dimension. The resulting ^1^ $t_{R}$ of this profile is the mean value of these times weighted by their heights. The projected profiles width ^1^ $\tau_{R}$ can be estimated from the variance of the distribution weighted by the heights.

The second dimension retention time ^2^ $t_{R}$ and peak width ^2^ $\tau_{R}$ can similarly be estimated by projecting the slices onto the second‐dimension axis and using the mean value of the ^2^ $t_{R}$ values weighted by their heights. As this projection onto the second dimension results in a narrow distribution, the calculation of a peak width would result in a smaller number than the peak widths of each sliced peak. Therefore, the peak width ^2^ $\tau_{R}$ is calculated as the mean value of the peak widths of the slices weighted by their height.

To evaluate the accuracy of simulated retention times and peak widths, root mean squared error (rmse) and root mean squared relative error (rmsre) were used. For peak width comparison, the full width at half maximum (FWHM) was estimated from experimental data using a half‐height method. In the second dimension, the FWHM was determined for each modulated slice, and the largest value was taken as the measured 2FWHM. In the first dimension, peak maxima of the individual slices were projected onto the first‐dimension time axis. As this projection typically yields only a limited number of data points per peak, the experimentally determined 1FWHM is subject to increased uncertainty. Details on the calculation methods are provided in the Supporting Information (Section S6).

#### 2.1.6 The Simulated Setup

In the presented example of a GC×GC system with a thermal modulator, the system itself is simple, consisting only of a series of column modules and two thermal modulator modules, see Figure 1. Only one path is possible, going from the injector through the two columns to the detector.

The following input data is needed:

- Dimensions of each capillary segment (length, internal diameter, film thickness) and the stationary phase
- Oven temperature program
- Inlet pressures at the different oven temperature plateaus (preferred over nominal column flow)
- Modulator settings ($t_{\mathrm{MP}}$, $t_{\mathrm{hot}}$, $t_{\mathrm{shift}}$, $T_{\mathrm{hot}}$ (as offset above oven temperature), $T_{\mathrm{cold}}$ (both temperatures have limited influence on the simulation, because of the simplified modulation modeling)
- Retention parameters of the solutes using the K‐centric three parameter model [38], the $d_{f}$/d ‐ratio of the system for which the parameters were estimated, see Supporting Information Section S3, and the CAS identifier. These can be found in Tables S4–S7.

### 2.2 Instrumentation

For validation of the simulation results, measurements were performed using a GC×GC‐ToF‐MS system, consisting of an Agilent GC 7890B gas chromatograph (Agilent Technologies, Palo Alto, USA) coupled to a BenchTOF DX mass spectrometer (Markes International Ltd., Bridgend, UK). Sample introduction was carried out using a PAL2 autosampler (CTC Analytics, Switzerland).

Thermal modulation was achieved using a consumable‐free ZX2 thermal modulator (Zoex Corporation, Houston, USA), positioned on the second‐dimension column. The hot jet duration was set to 350 ms, with a temperature offset of 25°C above the temperature program of the GC oven.

A 30 m × 0.25 mm × 0.25 µm ZB‐1 ms column (Phenomenex Ltd. Deutschland, Aschaffenburg, DE) was used for the first dimension, and a 2 m × 0.1 mm × 0.1 µm Stabilwax column (Restek Corporation, Bellefonte, PA, USA) was used for the second dimension. The first modulation point was located 0.3 m downstream of the second‐dimension column inlet, followed by a 0.9 m loop returning to the cold/hot‐jet for the second modulation point. The remaining 0.53 m within the GC oven and 0.24 m in the transfer line to the ToF‐MS were used for the second‐dimension separation. The effective length of each modulation point was measured as 5 mm. Based on hold‐up time measurements of air under various inlet pressures and oven temperatures (with modulation disabled), the length of the first‐dimension column was estimated as 29.74 m, see Supporting Information Section S4 and Table S8.

Helium was used as the mobile phase. The determined inlet pressures of 160.30 kPa(g) at 50°C and 274.96 kPa(g) at 225°C corresponded to a column flow of 0.8 mL/min defined by the control software TOF‐DS (Markes International Ltd., Bridgend, UK). According to the flow calculator [35] and using the system as defined in Figure 1, the real flow is 0.72 mL/min at 50°C and 0.78 mL/min at 225°C. The discrepancy between the set flow in the control software and the calculated flow is due to the limitation of defining the capillary system in the control software, which only allowed for two capillaries in series with the same temperature for all segments.

All temperature programs started at 50°C (held for 2 min) ramped up to 225°C. Different heating rates of 3°C/min, 5°C/min, and 10°C/min were used, together with different modulation periods of 3, 4, and 6 s, see Table 1. All measured and predicted retention times and peak widths can be found in the Supporting Information.

**TABLE 1:** Overview of the tested heating rates and modulation periods. The holding time of the final temperature varied. All other settings were the same for all measurements.

Measurement	Heating rate in°C/min	$t_{\mathrm{MP}}$ in s	$t_{\mathrm{end}}$ in min
I‐3‐3	3	3	10
II‐5‐3	5	3	15
III‐10‐3	10	3	15
IV‐3‐6	3	6	25
V‐3‐4	3	4	25
VI‐5‐4	5	4	5
VII‐3‐3	3	3	25
VIII‐5‐3	5	3	25

### 2.3 Chemicals

A mixture of fatty acid methyl esters standard (FAMEs) containing 37 FAMEs from C4:0 to C24:0 and concentrations between 200 and 600 µg/mL per component in *n*‐hexane and primary alcohols (heptanol, nonanol, decanol, undecanol), a 2‐alkanones‐mix (hexanone, decanone, undecanone, dodecanone, tridecanone, pentadecanone), and a *n*‐alkyl‐phenone‐mix (C_3_ ‐ C_7_) was used. All analytes had a purity of >98% and were purchased from Sigma–Aldrich. Further details can be found in Section S3 of the Supporting Information.

### 2.4 Software

GC×GC data analysis was performed using ChromSpace (Markes International Ltd., Bridgend, UK). The simulation was implemented in the Julia programming language [39], and GC×GC separations were simulated using the presented approach, published as the Julia package GasChromatographySystems.jl [36].

Retention parameters for the analytes on both columns were estimated using the Julia package RetentionParameterEstimator.jl [40] and can be found in the Supporting Information as well as in the retention database [34, 41]. Additional evaluations, including estimation of peak widths in the first and second dimensions and determination of the correct modulation period, were carried out using in‐house Julia scripts developed for this study.

## 3 Results and Discussion

### 3.1 Initial Results and Modulation Period Correction

**FIGURE 2:**
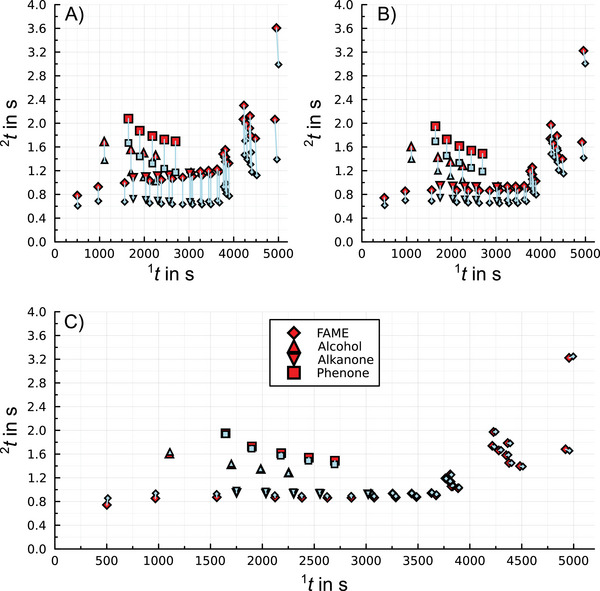
Example of measured (red) and predicted (light blue) two‐dimensional retention times, connected by a blue line, for measurement V‐3‐4 (heating rate of 3°C/min, assumed modulation period of 4 s). A) Using nominal parameters of the GC×GC system. B) Correction of the modulation period to 4.000309 s. C) Additional shift of the modulation by 0.24 s. This is the final result.

Predicting the two‐dimensional retention times using the described simulation method and the nominal parameters of the GC×GC system results in a pattern resembling the measured chromatogram, see Figure 2A. While the first‐dimension retention times match reasonably well (rmse(^1^ $t_{R}$) = 5.1 s to 17.4 s, rmrse(^1^ $t_{R}$) 0.3% to 0.8%, see first column Table 3), second‐dimension retention times deviate significantly (rmse(^2^ $t_{R}$) 330 to 520 ms, rmsre(^2^ $t_{R}$) 34% to 38%, see first column Table 3). In general, an increasing deviation Δ ^2^ $t_{R}=$ ^2^ $t_{R,\mathrm{meas}}-$ ^2^ $t_{R,\mathrm{sim}}$ with increasing ^1^ $t_{R}$ can be observed, see Figure S6A. This leads to the hypothesis of an increased actual value of the modulation period.

Isothermal measurements at an elevated oven temperature of 200°C were used to measure the deviation in retention times of the column bleed from the first‐dimension column, see Supporting Information Section S5 and Figures S2–S5. The corrected modulation periods are listed in Table 2. Though the correction is less than a millisecond, the accumulated deviation after 1000 modulations is in the same range as the observed errors, for example, 0.309 s for an assumed modulation period of 4 s.

**TABLE 2:** Estimated actual modulation periods.

Assumed $t_{\mathrm{MP},\mathrm{set}}$ in s	Actual $t_{\mathrm{MP},\mathrm{corr}}$ in s
3.0	3.000236 ± 0.5e‐6
4.0	4.000309 ± 0.7e‐6
6.0	6.000461 ± 1.3e‐6

### 3.2 Modulation Shift Corrections

After re‐evaluating the data with the corrected modulation period, the similarity between measured and predicted chromatograms improved moderately, Figure 2B. Deviations in ^1^ $t_{R}$ improved slightly, while for ^2^ $t_{R}$ the improvement is significant but still not satisfying. Overall, the rmse(^2^ $t_{R}$) values improved roughly by a factor of 2, from a range of 327 to 522 ms originally (see first column Table 3) to a range of 209 to 278 ms (see second column Table 3). These results align with prediction errors in previously reported studies [15, 18, 19, 20, 22].

**TABLE 3:** Root mean squared errors (rmse) of ^1^ $t_{R}$ (in s) and ^2^ $t_{R}$ (in ms). In brackets, the corresponding root mean squared relative errors (rmsre) are noted. Alternative metrics of normalized Euclidean distance, Table S9, and for normalized cosine similarity, Table S10, can be found in the Supporting Information.

Measurement	Original		+ $t_{\mathrm{MP}}$ corr.		+ $t_{\mathrm{shift}}$ corr.	
	rmse(^1^ $t_{R}$) in s	rmse(^2^ $t_{R}$) in ms	rmse(^1^ $t_{R}$) in s	rmse(^2^ $t_{R}$) in ms	rmse(^1^ $t_{R}$) in s	rmse(^2^ $t_{R}$) in ms
I‐3‐3	9.52 (0.29%)	522 (37%)	9.36 (0.27%)	278 (24%)	9.26 (0.27%)	55.1 (4.1%)
II‐5‐3	11.1 (0.44%)	405 (36%)	9.27 (0.36%)	225 (24%)	9.14 (0.36%)	38.5 (5.1%)
III‐10‐3	12.5 (0.79%)	327 (36%)	10.8 (0.67%)	209 (26%)	10.6 (0.66%)	49.5 (8.0%)
IV‐3‐6	14.8 (0.49%)	473 (34%)	14.6 (0.46%)	227 (20%)	14.4 (0.44%)	33.3 (3.6%)
V‐3‐4	15.7 (0.48%)	496 (35%)	13.1 (0.42%)	231 (20%)	13.0 (0.41%)	32.6 (3.6%)
VI‐5‐4	5.07 (0.33%)	394 (38%)	4.04 (0.25%)	243 (27%)	3.98 (0.24%)	40.1 (4.7%)
VII‐3‐3	13.4 (0.42%)	516 (38%)	12.7 (0.38%)	264 (26%)	12.6 (0.37%)	50.0 (3.9%)
VIII‐5‐3	17.4 (0.65%)	424 (35%)	14.5 (0.53%)	229 (23%)	14.4 (0.52%)	39.7 (4.9%)

The deviation Δ ^2^ $t_{R}$ can be further reduced by using a constant shift between the injection time and the modulation using the average deviation of all analytes for all measurements, resulting in a shift of $t_{\mathrm{shift}}=$ 0.24 ± 0.04 s, see also Figure S6B. This shift may be due to constant delays from the hot‐jet control system and heat transfer latency.

Figure 2C shows the final result after applying all corrections. The rmse(^1^ $t_{R}$)‐value is reduced to 3.98 s to 14.4 s (0.24% to 0.66%) and for the second dimension rmse(^2^ $t_{R}$) is in the significantly reduced range of 32.6 ms to 55.1 ms (3.6% to 8.0%).

Jaramillo and Dorman [42] reported similar systematic ^2^ $t_{R}$ prediction errors and applied empirical corrections based on elution temperature and elution velocity to reduce the average prediction errors below 50 ms. The present study achieves comparable improvements through physical parameter corrections.

McGinitie and Harynuk [43] also observed systematic differences in the prediction of retention times when using the nominal column values. After they used a correction for measured column lengths and estimation of column diameters and film thickness based on matching predicted and measured retention times for selected solutes, Δ ^2^ $t_{R}$ was, on average, below 50 ms.

A further correction for film thickness, respectively, column diameters could improve the results further. An additional correction for diameters of the columns is partially already implemented by determining the length of the first dimension column using hold‐up time measurements and nominal diameters and measured length of the second dimension column, see Supporting Information Section S4. As the ratio of L/d is relevant for the retention times and hold‐up times deviations of the column diameters are incorporated in the estimated length of the first dimension column.

### 3.3 Systematic Deviations in Retention Time Predictions

**FIGURE 3:**
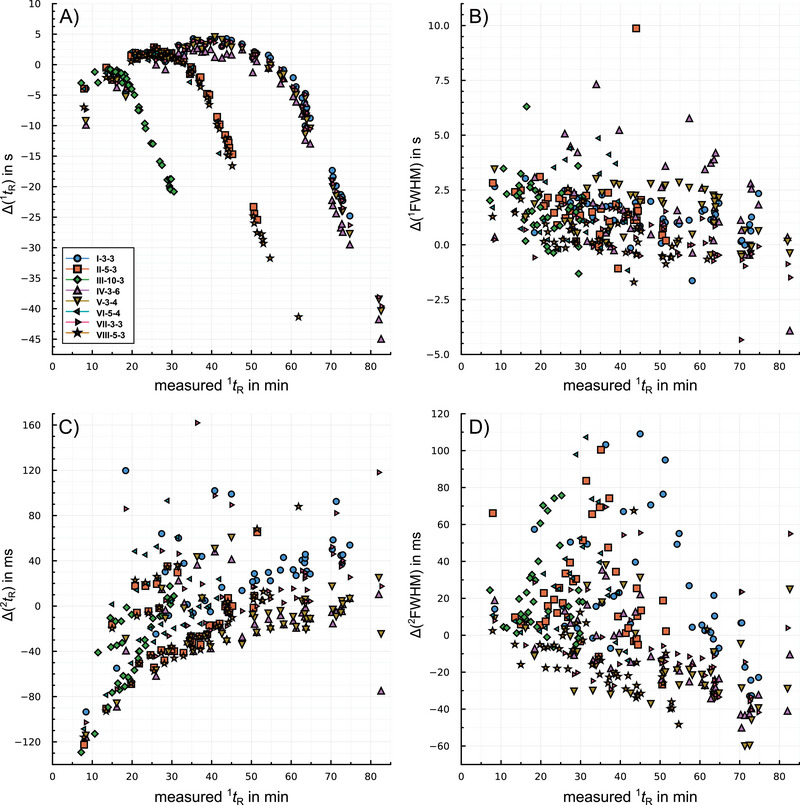
Retention time and peak width differences between measurements and predictions for all eight programs over the measured first dimension retention times. A) retention time difference in the first dimension Δ ^1^ $t_{R}$. B) peak width difference in first dimension Δ ^1^ FWHM. C) retention time difference in the second dimension Δ ^2^ $t_{R}$. D) peak width difference in second dimension Δ ^2^ FWHM.

Figure 3 shows the differences between measured and predicted retention times (Figure 3A for the first dimension and Figure 3C for the second dimension) and FWHM (Figure 3B in the first dimension, Figure 3D in the second dimension) in dependence on the measured first dimension retention time.

For the retention time difference in the first dimension, two deviations are observed. Δ ^1^ $t_{R}$ depends on the heating rates, with the higher values for the lowest heating rate of 3°C/min and the lower for the highest heating rate of 10°C/min. As the retention times increase with decreasing heating rate, the relative deviations are comparable for the different heating rates. Besides the influence of the heating rate, later eluting analytes show higher deviations in general (Pearson correlation coefficient ‐0.50 to ‐0.92 for different heating rates).

For ^2^ $t_{R}$, differences between measurements and predictions show a moderate increasing trend (Pearson correlation coefficient 0.44) in relation to ^1^ $t_{R}$ or across experimental conditions (Figure 3C). For the first eluting compound, Methyl butyrate, ^2^ $t_{R}$ is consistently predicted with a significantly higher value of 90 to 130 s than measured. Estimating retention parameters from temperature programmed measurements is less reliable for lower retained compounds [33, 44], as for such substances different used temperature programs result in similar retention times spanning only a small range of elution temperatures.

Reasons for the systematic differences of the predicted retention times in both dimensions are a combination of deviation of nominal values of the columns from actual values, in addition to errors in the estimated retention parameters. A slightly smaller film thickness could reduce the retention of the solutes, while a larger film thickness would increase the retention. A certain value of the film thickness, respectively, the phase ratio was assumed for the system where measurements for the estimation of retention parameters were made. For the GC×GC measurements, different columns were used. Slight differences in the phase ratios between the used columns lead to additional errors in the prediction. Also, deviations in the column diameters would introduce additional errors for the prediction. As mentioned before, this should partially be compensated for by the estimation of the first‐dimension column length from hold‐up time measurements, but this estimation does not account for the different diameter deviations for the two different columns. Further simulations with systematically varied ^1^ d, ^2^ d, ^1^ $d_{f}$, and ^2^ $d_{f}$ values were not performed, as this would be an unconstrained trial‐and‐error optimization without experimental justification. Addressing these deviations instead requires improved retention‐parameter estimation based on measurements performed on the same GC×GC system.

### 3.4 Prediction vs. Reproducibility

Measurements I‐3‐3 and VII‐3‐3, as well as II‐5‐3 and VIII‐5‐3, use identical GC programs except for the hold time at the end of the temperature program. Comparing ^1^ $t_{R}$ and ^2^ $t_{R}$ values between the two measurements allows a rough estimation of the uncertainty in the experimental data, which can then be compared to the accuracy of the predicted retention times by the simulation.

For measurements I‐3‐3 and VII‐3‐3 rmse(^1^ $t_{R}$) is 1.0 s and rmse(^2^ $t_{R}$) is 18.2 ms, while for II‐5‐3 and VIII‐5‐3, corresponding values are 1.2 s and 4.6 ms. In contrast, the deviations between predicted and measured retention times are significantly higher: 9 to 15 s for ^1^ $t_{R}$ and 39 to 55 ms for ^2^ $t_{R}$. This comparison indicates that the prediction errors, particularly in the first dimension, exceed the experimental reproducibility. This underscores the need for further refinement of the simulation, especially through the use of retention parameters estimated directly from measurements on the same GC×GC system.

### 3.5 Peak Width Predictions and Examples

Peak width predictions of the simulation were less accurate overall, as shown in Figures 3B and 3D. Deviations Δ ^1^ FWHM and Δ ^2^ FWHM are calculated as the difference between measured and predicted FWHM. These deviations show both over‐ and underestimation, whereby underestimations are dominant. No clear pattern can be observed, neither for the heating rates, modulation periods, nor substance classes.

Differences in ^2^ FWHM show a grouping, Figure 3D. Measurements I‐3‐3, II‐5‐3, III‐10‐3, and VI‐5‐4 tend to significantly broader measured peaks than predicted. Chromatograms of these measurements showed a stronger peak tailing, resulting in higher values of the measured ^2^ FWHM than for the other four measurements. Between the two groups of measurements was a period of about one week during which no measurements were made with the system. It is assumed that insufficient conditioning of the GC×GC system for the first set of measurements resulted in lower quality chromatograms.

**FIGURE 4:**
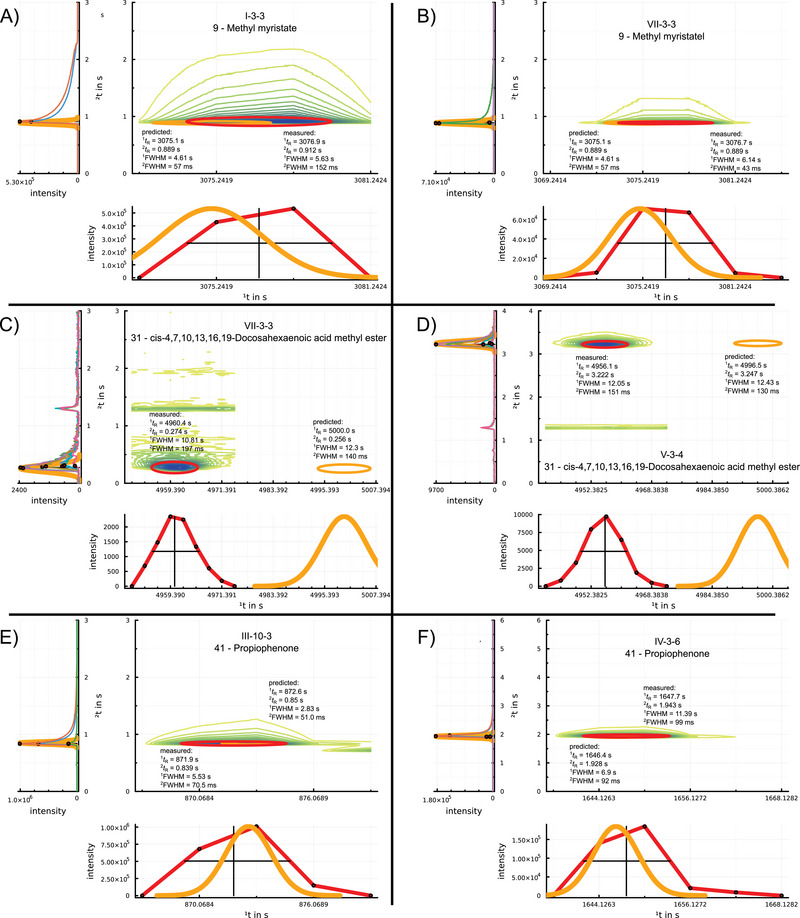
Details of the measured (red) and simulated (orange) peaks in both dimensions for a selection of three solutes of different measurements. In each example, the top left subplot shows the second dimension chromatograms of the slices of the analyte (different colored lines) and the apex of the analyte in each slice (red dot). The bottom right subplot shows the projection of the analyte apex onto the first dimension (red dots connected by red lines). The top right subplot shows the measured two‐dimensional chromatogram as a contour plot together with the ellipses indicating measured (red), respectively predicted (orange) peak widths and retention times in 2D. A) Methyl myristate from measurement I‐3‐3. B) Methyl myristate from measurement VII‐3‐3. C) cis‐4,7,10,13,16,19‐Docosahexaenoic acid methyl ester from measurement VII‐3‐3, with wraparound. D) cis‐4,7,10,13,16,19‐Docosahexaenoic acid methyl ester from measurement V‐3‐4, without wraparound. E) Propiophenone from measurement III‐10‐3. F) Propiophenone from measurement IV‐3‐6.

Figure 4A shows such a strong peak tailing of Methyl myristate for measurement I‐3‐3, while Figure 4B shows the peak of the same substance with less tailing for measurement VII‐3‐3 with the same settings. The predicted peak width of 57 ms is smaller than the measured 152 ms for I‐3‐3, while for VII‐3‐3 the same predicted peak width is larger than the measured peak width of 43 ms. This shows that external sources of peak broadening can have a significant influence on resulting peak widths, which are not addressed by the prediction model.

An overview of the prediction accuracy for peak widths is provided in Table 4. The rmse for ΔFWHM range between 1.0 – 2.7 s (20 – 36%) for the first dimension and 23.5 – 41.2 ms (31 – 56%) for the second dimension, confirming that peak width predictions show greater variability than retention time predictions.

**TABLE 4:** Root mean squared errors (rmse) of peak widths for both dimensions. In brackets, the corresponding root mean squared relative errors (rmsre) are noted.

Measurement	rmse(Δ ^1^ FWHM) in s	rmse(Δ ^2^ FWHM) in ms
I‐3‐3	1.5 (24%)	40.2 (33%)
II‐5‐3	2.3 (31%)	36.3 (33%)
III‐10‐3	2.2 (36%)	31.5 (31%)
IV‐3‐6	2.7 (28%)	25.2 (47%)
V‐3‐4	1.8 (24%)	27.8 (56%)
VI‐5‐4	2.2 (31%)	41.2 (36%)
VII‐3‐3	1.1 (23%)	27.1 (40%)
VIII‐5‐3	1.0 (20%)	23.5 (49%)

Figures 4C and 4D show the chromatographic behavior of cis‐4,7,10,13,16,19‐Docosahexaenoic acid methyl ester in measurements VII‐3‐3 and V‐3‐4, respectively. This late‐eluting FAME has an increased retention on the second‐dimension column, which results in wraparound effects for short modulation periods. Peak width prediction for the first‐dimension match measured values, while for the second dimension, the peak width is under‐predicted. This demonstrates that the simulation can predict wraparound peaks with a similar accuracy to non‐wrapped peaks.

Figures 4E and 4F show the peak of Propiophenone in measurements III‐10‐3 and IV‐3‐6. The predicted first‐dimension peak widths are significantly narrower than the measured ones, 2.8 s (predicted) vs. 5.5 s (measured) in Figure 4E, and 6.9 s (predicted) vs. 11.4 s (measured) in Figure 4F, showing an underestimation of dispersion for this solute. For these examples, sparse sampling of the measured peaks can contribute to an increased measured peak width. In contrast, the second‐dimension peak widths for these two examples agree more closely between simulation and measurement.

## 4 Conclusion

Using a modular and graph‐based simulation approach, predictions of retention times and peak widths for a complex thermal modulated GC×GC system could be made for measurements using different heating rates and modulation periods. Initial observed deviations could largely be explained and compensated for by adjusting parameters of the modulator (actual modulation period and the temporal offset of thermal modulation). Using these modifications, the agreement between simulation and experiment could be significantly improved.

The presented simulation demonstrates the significant impact of various system parameters on the resulting chromatogram in GC×GC. Even minor deviations, such as slight shifts in the actual modulation period or deviations, can lead to systematic changes in retention times. These effects are relevant in long‐running experiments, such as in metabolomics studies, where instrumental conditions can drift over time and distort chromatographic results, requiring correction during data analysis.

These findings highlight the potential of the simulation not only for method development but also for retrospective system diagnostics. Changes in column behavior over time, which affect separation performance, could be detected and understood through simulation‐based analysis.

In future developments, retention parameters should be estimated directly from GC×GC measurements, similar to established approaches in 1D‐GC [33]. This should further improve the accuracy by inherently accounting for system‐specific deviations like film thickness or column diameters from nominal values. This would allow simulations based on nominal hardware parameters to better reflect actual retention behavior. A similar approach was successfully demonstrated by Barcaru et al. [17], who achieved a second‐dimension retention time deviation of about 50 ms, comparable to the accuracy attained in this work after applying corrections.

Overall, the simulation provides a powerful and efficient tool for exploring, optimizing, and validating thermal modulated GC×GC systems. It paves the way toward more robust, reproducible analyses and could serve as a foundation for automated method development and quality control in multidimensional gas chromatography, by incorporating the prediction model in machine learning tools, for example, physical informed neural networks.

As the simulation uses a modular approach, predictions for other complex GC systems, for example, GC×GC×GC, can be made. The simulation model can easily be expanded to incorporate different modulation techniques, like flow modulation, as the working principle of periodic reinjection of segments from analytes eluting from a first column onto a second column is the same. The main difference would be in the difference in flow as the pressure at the modulation point is separately controlled, which can be modeled by the modular graph approach.

## Author Contributions

**Jan Leppert**: conceptualization, data curation, formal analysis, funding acquisition, investigation, methodology, project administration, software, supervision, validation, visualization, writing – original draft, writing – review and editing. **Tillman Brehmer**: Investigation, Methodology, Writing – original draft. **Matthias Wüst**: Resources, Supervision

## Conflicts of Interest

The authors declare no conflicts of interest.

## Declaration of Use of AI‐Assisted Technologies

In the development of this work, the author employed ChatGPT (OpenAI) to enhance the readability and language quality of the manuscript. Following the utilization of this tool, the authors reviewed and edited the material as necessary and take full responsibility for the content of the publication.

## Supporting information




**Supporting File 1**: jssc70351‐sup‐0001‐SuppMat.pdf.

**Supporting File 2**: jssc70351‐sup‐0002‐SuppMat.xlsx.

**Supporting File 3**: jssc70351‐sup‐0003‐SuppMat.xlsx.

**Supporting File 4**: jssc70351‐sup‐0004‐SuppMat.xlsx.

## Data Availability

The authors confirm that the data supporting the findings of this study are available within the article and its supplementary materials.
