# Supplementary material for: Prediction of Thermal Modulated Comprehensive Two‐Dimensional Gas Chromatographic Separation Using a Modular, Graph‐Based Simulation Platform
Source: J Sep Sci. 2026 Jan 21;49(1):e70351. doi: 10.1002/jssc.70351 (PMC12824439; doi:10.1002/jssc.70351)
Supplement: Supplementary file 1 — Supporting File 1: jssc70351‐sup‐0001‐SuppMat.pdf. [file JSSC-49-e70351-s001.pdf]

Supplementary Material

Prediction of thermal modulated comprehensive two-dimensional gas chromatographic separation using a modular, graph-based simulation platform

Jan Leppert*, Tillman Brehmer, Matthias Wüst

University of Bonn, Institute of Nutritional and Food Sciences, Chair of Food Chemistry, Friedrich-Hirzebruch-Allee 7, 53115 Bonn, Germany

*Corresponding author: jleppert@uni-bonn.de

Tabel of content

S.1 List of symbols 3

S.2 Definition of modules 4

S.3 Retention parameter estimation 6

S.4 Hold-up time measurements and length estimation 10

S.5 Estimation of actual modulation period 11

S.6 Additional metrics 14

Root mean squared error 14
Full width at half maximum 14
Normalized Euclidean Distance in Two-Dimensional Retention Time Space 14
Normalized Cosine Similarity of Retention Time Patterns 15

S.7 Measured and predicted retention times and peak widths 17

S.8 Code examples 18

S.9 Pluto notebook 22

S.10 References 23

S.1 List of symbols

Table S1. List of symbols.

Symbol	Description
L	Length of a column, capillary or module
d	Internal diameter of a column or capillary
d_{f}	Film thickness of the stationary phase in a column
F	Flow through a column, capillary or module
t_{MP}	Modulation period
t_{shift}	Shift of the modulation in relation to the injection time
t_{cold}	Time of active cold jet
t_{hot}	Time of active hot jet
T_{hot}	Relative temperature increase during active hot jet
T_{cold}	Temperature of the cold jet
T	Temperature, often as oven temperature
p	Pressure
x	Point along a column, capillary or module
t	Time
i	Index number of a module
p_{in}	Inlet pressure
p_{out}	Outlet pressure
s	Index number of a substance
t_{init}	Initial time resp. start time
τ_{init}	Initial peak width (standard deviation)
t_R	Retention time
τ_R	Peak width (standard deviation) at t_R
$t_{\mathrm{next\ hot}}$	Time of the start of the next hot jet
$t_{\mathrm{migration}}$	Time to migrate through a column, capillary or module
u_{R}	Solute velocity
u_{M}	Mobile phase velocity
k	Retention factor
K	Distribution coefficient
β	Phase ratio
φ	Dimensionless film thickness
rmse	Root mean squared error
rmsre	Root mean squared relative error
${}^1t_{\mathrm{R}}, {}^2t_{\mathrm{R}}$	retention times of 1st and 2nd dimension
$\Delta {}^1t_{\mathrm{R}}, \Delta {}^2t_{\mathrm{R}}$	Difference of measured and predicted retention times of 1st and 2nd dimension
${}^1\tau_{\mathrm{R}}, {}^2\tau_{\mathrm{R}}$	peak width (standard deviation) of 1st and 2nd dimension
${}^1FWHM, {}^2FWHM$	Full widths at half maximum (FWHM) for 1st and 2nd dimension
$\Delta {}^1FWHM, \Delta {}^2FWHM$	Difference of measured and predicted FWHM of 1st and 2nd dimension

S.2 Definition of modules

Table S2. Structure of the column module `ModuleColumn`.

Parameter	Description
**name**	name of the module
**L**	length of the column in m
**d**	internal diameter of the column in mm
**df**	film thickness of the column in μm
**sp**	name of the stationary phase
**T**	temperature of the column, can be a number or a function, in °C
**F**	flow through the column in mL/min, if it is unknown and should be calculated its value is NaN (not a number)
**opt**	additional options

Table S3. Structure of the module `ModuleTM`.

Parameter	Description
**name**	name of the module
**L**	length of the modulation column in m
**d**	diameter of the modulation column in mm
**df**	film thickness of the modulation column in μm
**sp**	name of the stationary phase
**T**	temperature of the oven around the modulation column, can be a number or a function, in °C
**shift**	shift of the modulation against the injection in s
**PM**	modulation period in s
**ratio**	the ratio of the time of active cold jet to active hot jet
**Thot**	the relative increase of the temperature while hot jet is active in °C. $T_{\mathrm{oven}} + T_{\mathrm{hot}}$
**Tcold**	the absolute temperature of the cold jet (if option `Tcold_abs = true`) or the relative change of temperature while cold jet is active (if option `Tcold_abs = false`), in °C. T_{cold}. resp $T_{\mathrm{oven}} + T_{\mathrm{cold}}$
**F**	flow through the column in mL/min, if it is unknown and should be calculated its value is NaN (not a number)
**opt**	additional options, like `Tcold_abs`

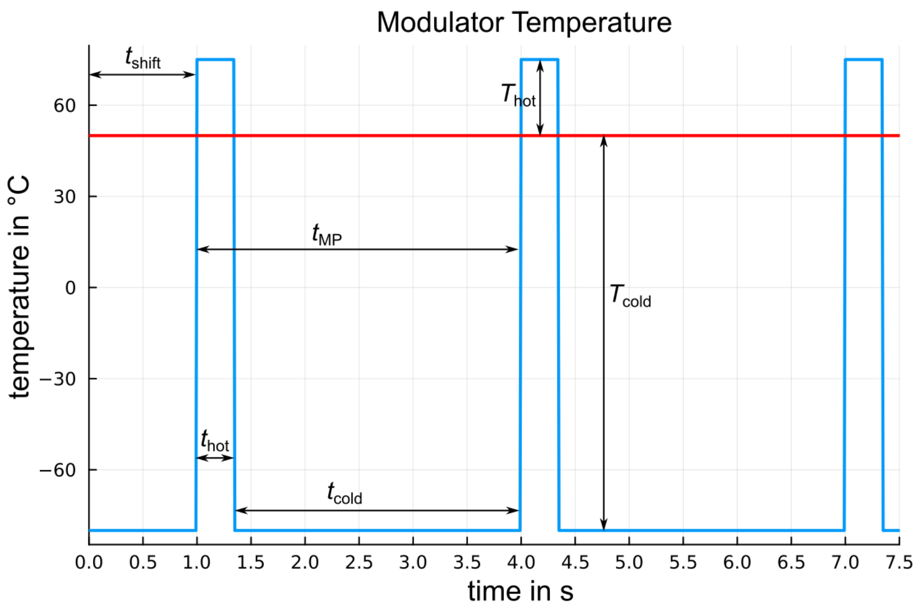

Figure S1. Periodic temperature modulation (blue) and temperature program of the GC oven (red).

S.3 Retention parameter estimation

To estimate the retention parameters of the solutes used for the evaluation of the GC×GC prediction, equal columns as in the GC×GC system were used in a conventional 1D-GC. A ZB1ms column ($L = 29.6 \pm 0.3$ m, $d = 0.25$ mm, $d_{\mathrm{f}} = 0.25$ µm, Phenomenex Ltd. Deutschland, Aschaffenburg, DE) and a Stabilwax column ($L = 10.3 \pm 0.5$ m, $d = 0.1$ mm, $d_{\mathrm{f}} = 0.1$ µm, Restek Corporation, Bellefonte, PA, USA). The column lengths, respectively the L/d-ratios, were estimated by hold-up time measurements. An Agilent GC 7890A gas chromatograph (Agilent Technologies, Palo Alto, USA) and a BenchToF mass spectrometer (Markes International Ltd., Bridgend, UK) together with a CTC PAL3 autosampler (CTC Analytics, Switzerland) were used. This system is different from the GC×GC system.

Each mix of FAMEs, primary alcohols, 2-alkanones, and phenones were measured with different temperature programs. The retention times of the solutes were measured. Using the open-source Julia package RetentionParameterEstimator.jl [1] the retention parameters were estimated from the measured retention times of the different temperature programs using the simulation GasChromatographySimulator.jl, by finding the set of retention parameters which minimizes the differences between measured and predicted retention times [2]. Methode 'm2' was applied, in which the three retention parameters of each analyte together with the L/d-ratio of the column is optimized.

For the Stabilwax column the following six temperature programs were used: starting with 40°C, immediately heating up with different heating rates to 250°C and holding this temperature for 10 min. The six heating rates were 3, 5, 10, 15, 20, and 30°C/min. The inlet pressure at 40°C was 440.194 kPa(g) and at 250°C it was 732.025 kPa(g), resulting in a flow of approximately 1.0 mL/min.

For the ZB1ms column the following five temperature programs were used: starting with 40°C, immediately heating up with different heating rates to 300°C and holding this temperature for 10 min. The five heating rates were 3, 5, 10, 15, and 20°C/min. The inlet pressure at 40°C was 48.745 kPa(g) and at 300°C it was 148.024 kPa(g), resulting in a flow of approximately 1.0 mL/min.

The results of the estimated retention parameters for both columns can be found in table S4 and S5.

For the ZB1ms column the estimated L/d-ration using the RetentionParameterEstimator.jl is estimated as 120000 ± 3000, which showed good agreement with the previously measured ratio using hold-up times of 118000 ± 1000. For the Stabilwax column the L/d-ratio from the RetentionParameterEstimator.jl was estimated with a larger uncertainty as 113000 ± 12000, while using hold-up time measurements the ratio was 103000 ± 5000.

The data of the measured retention times can be found in the Excel files:

- '**SuppMat_RPE_measuredRT_Stabilwax.xlsx**'
- '**SuppMat_RPE_measuredRT_ZB1ms.xlsx**'

Table S4. Retention parameters for the ZB1ms column, dimensionless film thickness $d_f/d = 0.001$, sorted by increasing T_{char} within each solute group.

No	Name	CAS	T_{char} [°C]	θ_{char} [°C]	ΔC_p [Jmol^{-1}K^{-1}]
**1**	Methyl butyrate	623-42-7	53.8	27.0	91.9
**2**	Methyl hexanoate	106-70-7	95.7	29.5	87.3
**3**	Methyl octanoate	111-11-5	131.7	31.3	89.9
**4**	Methyl decanoate	110-42-9	163.1	32.6	93.9
**5**	Methyl undecanoate	1731-86-8	177.4	33.3	100.8
**6**	Methyl laurate	111-82-0	190.8	33.8	105.0
**7**	Methyl tridecanoate	1731-88-0	203.5	34.3	110.3
**8**	Methyl myristoleate	56219-06-8	214.2	35.0	110.2
**9**	Methyl myristate	124-10-7	215.5	34.7	114.5
**10**	Methyl cis-10-pentadecenoate	90176-52-6	225.8	35.5	116.8
**11**	Methyl pentadecanoate	7132-64-1	227.0	35.2	120.6
**12**	Methyl palmitoleate	1120-25-8	235.8	35.8	116.6
**13**	Methyl palmitate	112-39-0	237.8	35.5	124.5
**14**	cis-10-Heptadecenoic acid methyl ester	75190-82-8	246.3	36.1	122.2
**15**	Methyl heptadecanoate	1731-92-6	248.2	35.9	130.2
**16**	Methyl gamma-linolenate	16326-32-2	253.9	36.9	122.3
**17**	Methyl linolelaidate	2566-97-4	255.1	36.4	117.7
**19**	Methyl linoleate	112-63-0	255.8	36.8	121.8
**18**	Methyl linolenate	301-00-8	255.2	36.2	130.0
**20**	trans-9-Elaidic acid methyl ester	1937-62-8	255.9	36.5	125.7
**21**	cis-9-Oleic acid methyl ester	112-62-9	256.1	36.3	127.6
**22**	Methyl stearate	112-61-8	258.1	36.2	135.2
**23**	cis-5,8,11,14,17-Eicosapentaenoic acid	2566-89-4	271.3	37.7	126.8
**24**	cis-5,8,11,14-Eicosatetraenoic acid methyl	2734-47-6	271.7	37.7	122.5
**25**	cis-11,14,17-Eicosatrienoic acid methyl ester	55682-88-7	272.6	37.4	129.1
**26**	cis-11,14-Eicosadienoic acid methyl ester	2463-02-7	274.2	37.3	136.0
**27**	cis-8,11,14-Eicosatrienoic acid methyl ester	21061-10-9	274.7	37.4	134.5
**28**	Methyl cis-11-eicosenoate	2390-09-2	274.4	36.9	130.4
**29**	Methyl arachidate	1120-28-1	276.5	36.8	143.7
**30**	Methyl heneicosanoate	6064-90-0	285.1	36.9	146.1
**31**	cis-4,7,10,13,16,19-Docosahexaenoic acid	301-01-9	288.1	38.5	135.8
**32**	1-Heptanol	111-70-6	104.5	30.4	136.9
**33**	1-Nonanol	143-08-8	139.8	32.1	110.7
**34**	1-Decanol	112-30-1	155.7	32.9	111.1
**35**	1-Undecanol	112-42-5	170.7	33.5	112.2
**36**	2-Decanone	693-54-9	143.2	32.4	91.8
**37**	2-Undecanone	112-12-9	159.0	33.1	94.5
**38**	2-Dodecanone	6175-49-1	173.7	33.7	97.7
**39**	2-Tridecanone	593-08-8	187.6	34.3	103.0
**40**	2-Pentadecanone	2345-28-0	213.1	35.1	108.8
**41**	Propiophenone	93-55-0	139.2	34.0	79.5
**42**	Butyrophenone	495-40-9	153.5	34.6	83.3
**43**	Valerophenone	1009-14-9	169.1	35.1	83.5
**44**	Hexanophenone	942-92-7	183.8	35.7	91.0
**45**	Heptanophenone	1671-75-6	197.6	36.2	93.9

Table S5. Retention parameters for the Stabilwax column, dimensionless film thickness $d_f/d = 0.001$, sorted by increasing T_{char} within each solute group. Numbers follow the order of analytes from Table S4.

No	Name	CAS	T_{char} [°C]	θ_{char} [°C]	ΔC_p [Jmol⁻¹K⁻¹]
**1**	Methyl butyrate	623-42-7	47.8	20.2	-204.0
**2**	Methyl hexanoate	106-70-7	82.0	22.7	-48.8
**3**	Methyl octanoate	111-11-5	113.2	24.7	-5.2
**4**	Methyl decanoate	110-42-9	141.3	26.3	19.5
**5**	Methyl undecanoate	1731-86-8	154.3	26.9	25.9
**6**	Methyl laurate	111-82-0	166.7	27.5	32.9
**7**	Methyl tridecanoate	1731-88-0	178.6	28.1	40.5
**9**	Methyl myristate	124-10-7	189.9	28.6	46.0
**8**	Methyl myristoleate	56219-06-8	194.2	29.0	40.8
**11**	Methyl pentadecanoate	7132-64-1	200.8	29.1	52.3
**10**	Methyl cis-10-pentadecenoate	90176-52-6	205.1	29.6	49.3
**13**	Methyl palmitate	112-39-0	211.2	29.6	59.0
**12**	Methyl palmitoleate	1120-25-8	214.2	30.0	51.0
**15**	Methyl heptadecanoate	1731-92-6	221.2	30.0	65.0
**14**	cis-10-Heptadecenoic acid methyl ester	75190-82-8	224.3	30.6	61.3
**22**	Methyl stearate	112-61-8	230.9	30.6	74.1
**20**	trans-9-Elaidic acid methyl ester	1937-62-8	233.9	31.6	76.1
**21**	cis-9-Oleic acid methyl ester	112-62-9	233.3	31.0	70.6
**17**	Methyl linolelaidate	2566-97-4	238.0	31.7	71.4
**19**	Methyl linoleate	112-63-0	237.4	31.0	67.6
**16**	Methyl gamma-linolenate	16326-32-2	241.0	32.0	68.7
**18**	Methyl linolenate	301-00-8	244.2	32.2	76.0
**29**	Methyl arachidate	1120-28-1	249.6	31.9	97.1
**28**	Methyl cis-11-eicosenoate	2390-09-2	252.0	32.5	90.3
**26**	cis-11,14-Eicosadienoic acid methyl ester	2463-02-7	256.9	33.4	102.2
**25**	cis-11,14,17-Eicosatrienoic acid methyl ester	55682-88-7	259.9	34.0	102.7
**30**	Methyl heneicosanoate	6064-90-0	259.0	33.1	120.3
**23**	cis-5,8,11,14-Eicosatetraenoic acid methyl	2566-89-4	262.0	34.1	94.4
**27**	cis-8,11,14-Eicosatrienoic acid methyl ester	21061-10-9	263.5	34.6	118.9
**24**	cis-5,8,11,14,17-Eicosapentaenoic acid	2734-47-6	269.3	36.1	126.2
**31**	cis-4,7,10,13,16,19-Docosahexaenoic acid	301-01-9	291.3	40.3	210.7
**32**	1-Heptanol	111-70-6	120.4	23.9	-10.9
**33**	1-Nonanol	143-08-8	147.5	25.4	14.3
**34**	1-Decanol	112-30-1	160.1	26.1	25.8
**35**	1-Undecanol	112-42-5	172.0	26.7	33.3
**36**	2-Decanone	693-54-9	128.2	25.7	-5.9
**37**	2-Undecanone	112-12-9	142.1	26.4	4.3
**38**	2-Dodecanone	6175-49-1	155.4	27.2	15.8
**39**	2-Tridecanone	593-08-8	168.0	27.8	24.4
**40**	2-Pentadecanone	2345-28-0	191.6	29.0	41.5
**41**	Propiophenone	93-55-0	160.3	29.5	11.0
**42**	Butyrophenone	495-40-9	168.9	29.9	17.0
**43**	Valerophenone	1009-14-9	181.0	30.4	22.5
**44**	Hexanophenone	942-92-7	192.8	30.9	27.0
**45**	Heptanophenone	1671-75-6	204.3	31.5	35.9

Table S6. Additional retention parameters for analytes not used in the publication. ZB1ms stationary phase, dimensionless film thickness $d_f/d = 0.001$, sorted by increasing T_{char}.

**Name**	**CAS**	T_{char} [°C]	θ_{char} [°C]	ΔC_p [Jmol⁻¹K⁻¹]
2-Pentanone, 4-methyl-	108-10-1	57.5	27.7	118.1
1-Pentanol	71-41-0	64.6	26.3	-65.3
2-Hexanone	591-78-6	67.9	28.3	121.2
Octanophenone	1674-37-9	210.7	36.6	99.3
cis-13,16-Docosadienoic acid methyl ester	61012-47-3	291.6	37.9	150.5
Methyl erucate	1120-34-9	291.7	37.5	142.9
Methyl-Docosanoat	929-77-1	293.5	37.3	155.6
Methyl tricosanoate	2433-97-8	301.4	37.5	157.7
Methyl nervonate	2733-88-2	307.7	38.0	155.9
Methyl lignocerate	2442-49-1	309.1	37.6	161.2

Table S7. Additional retention parameters for analytes not used in the publication. ZB1ms stationary phase, dimensionless film thickness $d_f/d = 0.001$, sorted by increasing T_{char}.

**Name**	**CAS**	T_{char} [°C]	θ_{char} [°C]	ΔC_p [Jmol⁻¹K⁻¹]
2-Pentanone, 4-methyl-	108-10-1	55.4	21.9	-233.7
2-Hexanone	591-78-6	66.2	21.8	-169.1
1-Pentanol	71-41-0	90.5	21.9	-67.1
Octanophenone	1674-37-9	215.3	31.9	40.0
Methyl-Docosanoat	929-77-1	268.8	35.0	154.1
Methyl erucate	1120-34-9	271.6	35.9	152.0
cis-13,16-Docosadienoic acid methyl ester	61012-47-3	276.8	37.2	165.8
Methyl tricosanoate	2433-97-8	278.8	37.4	192.8
Methyl lignocerate	2442-49-1	288.7	39.8	223.3
Methyl nervonate	2733-88-2	293.2	41.9	198.7

S.4 Hold-up time measurements and length estimation

The length of the second-dimension column was measured as a total of 1.98 ± 0.02 m. The first modulation point is located 0.3 ± 0.01 m after the connection with the first-dimension column. The loop is 0.9 ± 0.01 m long. The remaining lengths are 0.53 ± 0.03 m in the GC oven and 0.24 ± 0.01 m in the heated transfer line to the TOF-MS. The lengths of the modulation points are measured as 0.005 ± 0.001 m.

To estimate the column length of the first dimension, multiple hold-up time measurements at different temperatures and inlet pressures were made, injecting dichloromethane and air. The air signal (m/z = 32) was used to measure the hold-up time. The thermal modulator was disabled for these measurements.

Table S8. Measured hold-up times t_M for different oven temperatures T_oven and inlet pressures p_inj.

$\boldsymbol{T_\mathrm{oven}}$ in °C	$\boldsymbol{p_\mathrm{inj}}$ in kPa(abs)	$\boldsymbol{t_\mathrm{M}}$ in s
**50**	206.8	346.77
**50**	258.8	279.85
**50**	258.8	279.23
**50**	258.8	279.49
**50**	306.8	236.79
**50**	370.0	197.33
**100**	233.4	324.17
**100**	295.2	259.17
**100**	346.2	223.05
**100**	417.5	186.69
**150**	259.4	302.01
**150**	328.1	243.41
**150**	384.8	210.27
**150**	464.0	175.79
**200**	284.9	276.31
**200**	360.3	224.93
**200**	422.6	195.23
**200**	509.7	165.03

A simplified system is modeled with GasChromatographySystems.jl consisting of three segments, with the first-dimension column in the oven, 1.74 m of the second-dimension column in the oven and 0.24 m of the second-dimension column in the transfer line at 250°C. Using the measured hold-up times at different oven temperatures and inlet pressures the length of the first-dimension column is calculated using a Nelder-Mead optimization to match measured and calculated hold-up times t_M.

The found optimum for the length of the first-dimension column is 29.74 ± 0.04 m.

S.5 Estimation of actual modulation period

Trying to match simulated GC×GC retention times with measured ones showed a systematic shift of ${}^{2}t_{\mathrm{R}}$ with increase of ${}^{1}t_{\mathrm{R}}$. This is partly a result of a deviation of the actual modulation period from the assumed value. For the used modulator it is a commonly known phenomenon, that due to aging of the crystal oscillator, used as timer, the actual modulation period increases slightly over time.

To measure this shift, isothermal measurements for 60 minutes with active modulation at 200°C, for the three modulation periods of 3 s, 4 s and 6 s, were made only injecting solvent (dichloromethane). Traces for the mass fragments 207, 267 and 281 were used to investigate the modulation of the column bleed of the first-dimension column. By utilizing a Julia programming language script that applies Savitzky-Golay smoothing with a window width of 17 points, the retention time of the modulated column bleed is estimated for each modulation. The shift in these retention times over the measurement runtime allows for the estimation of the actual modulation period through linear regression.

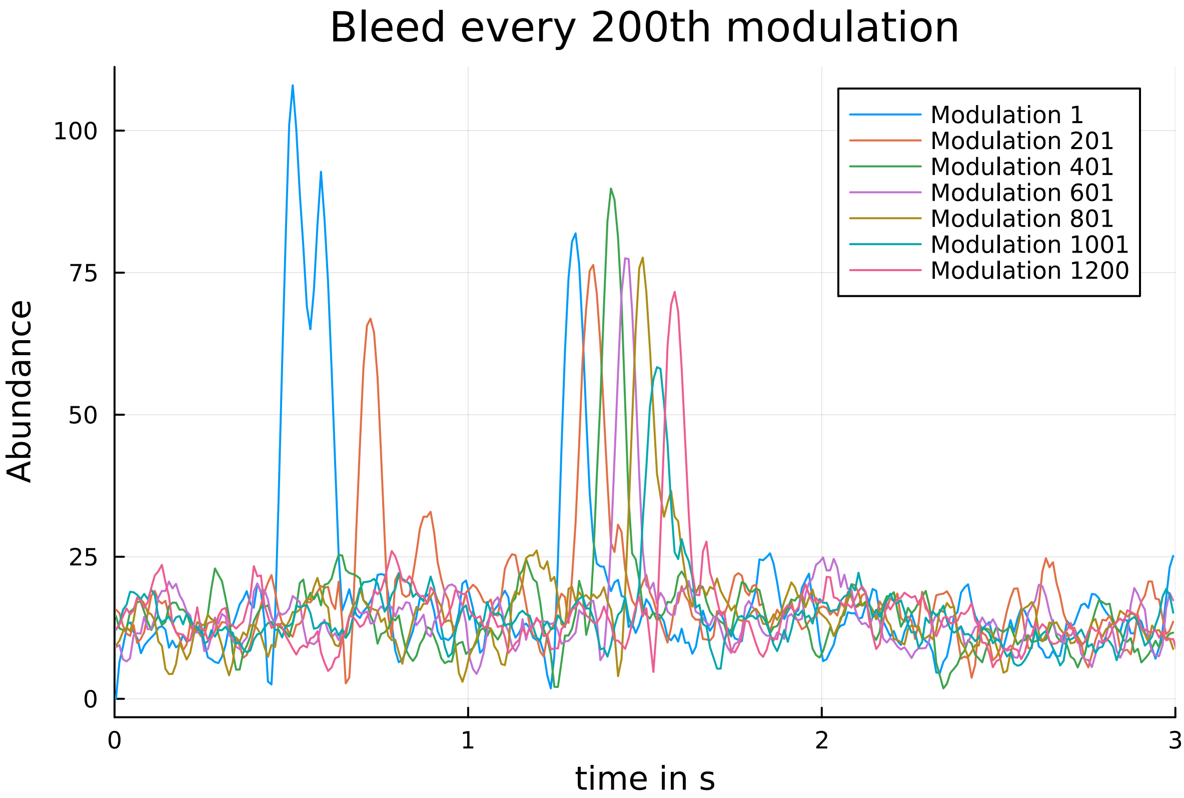

Figure S2. Trace of the column bleed (m/z = 207 + 267 + 281) for the assumed modulation period t_{MP} = 3 s and isothermal temperature of 200°C. A Savitzky-Golay smoothing with a window width of 17 points was used.

Figure S2 shows the peak of the column bleed for every 200th modulation period assuming a modulation period of 3 s at an oven temperature of 200°C. A clear shift of the ${}^{2}t_{\mathrm{R}}$ can be observed from 1.30 s at the first modulation to 1.58 s at 1200th modulation. For modulation periods of 4 s and 6 s a similar shift over the total measurement time of 60 min was observed. The observed additional earlier peaks for modulation 1 and 201 are not related to the continuous occurring column bleed (which results in a continuous band, see Figure S4).

Fitting a linear model ${}^{2}t_{\mathrm{R}} = {}^{2}t_{\mathrm{R},0} + m \cdot n$, where n is the number of the modulation, gives the shift of the actual modulation period from the assumed one, Figure S3. The actual modulation period is the sum of the assumed value and the slope m. The estimated actual modulation periods are listed in table 2.

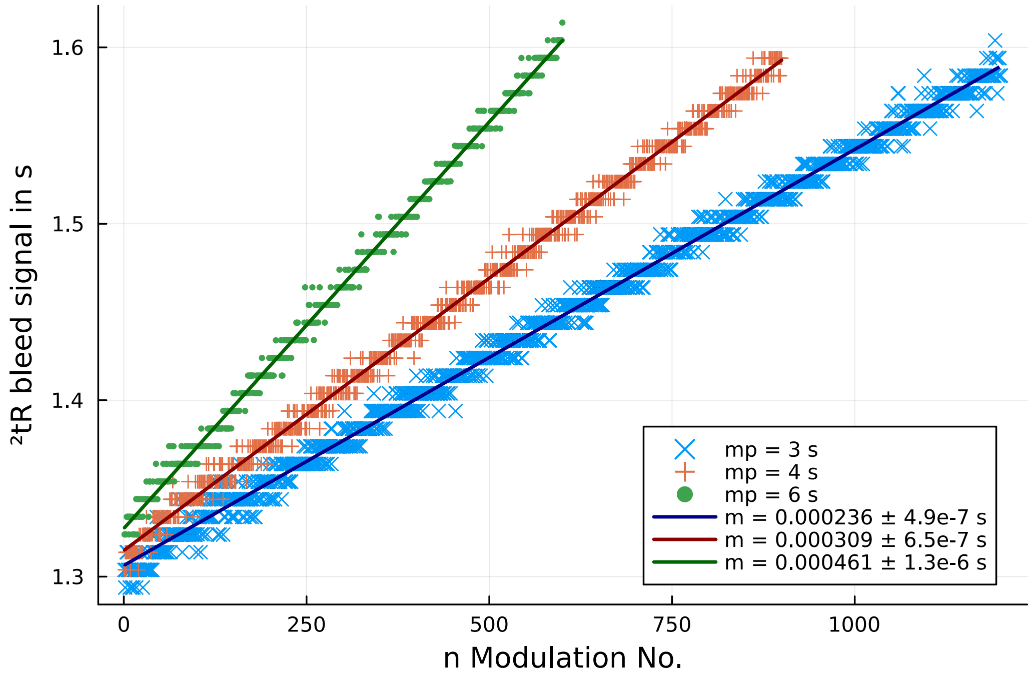

Figure S3. Second-dimension retention time of the column bleed plotted over the number ot the modulation, includind the linear fit.

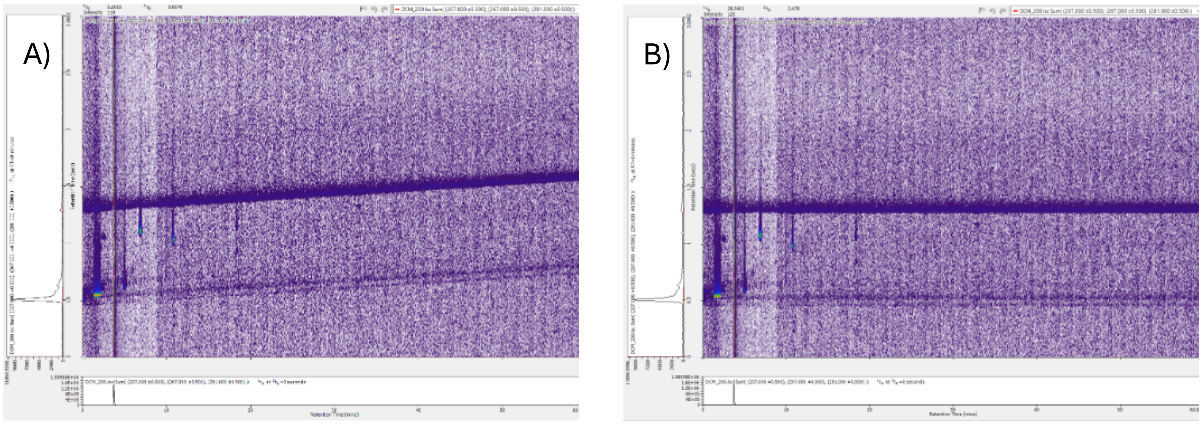

Figure S4. 2D chromatograms (m/z = 207 + 267 + 281) of isothermal measurement with A) nominal modulation period of 3 s and B) with corrected modulation period of 3.000236 s.

It is not possible to estimate the actual modulation period by comparing the differences between neighboring peaks of the column bleed, see Figure S4. The limited measurement rate and the small difference of actual and assumed modulation periods, results in small time difference of less than 1 ms. To measure such small differences a measurement rate of more than 1 kHz would be needed.

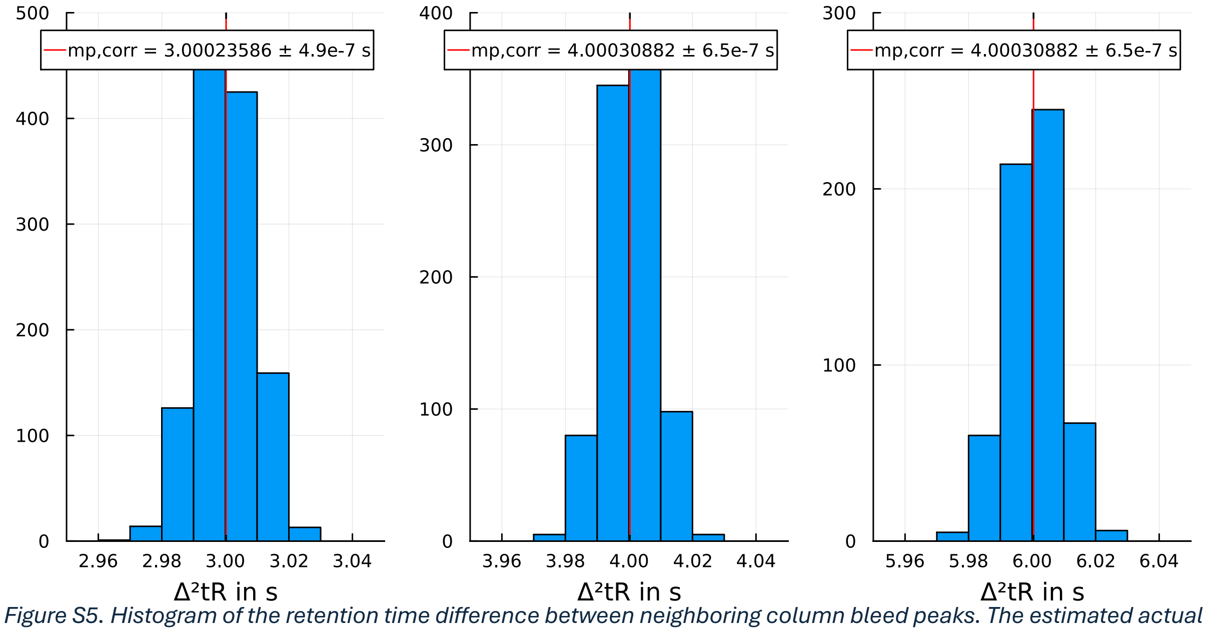

Figure S5. Histogram of the retention time difference between neighboring column bleed peaks. The estimated actual modulation period is marked with a red line.

S.6 Additional metrics

Root mean squared error

The following metrics are used to compare measured and predicted chromatograms by their two-dimensional retention times respectively peak widths.

The root mean squared error (rmse):

$$\mathrm{rmse(X)} = \sqrt{\frac{1}{n}\sum_{i=1}^{n}\left(X_{\mathrm{meas},i} - X_{\mathrm{sim},i}\right)^2} \tag{S1}$$

The root mean squared relative error (rmsre):

$$\mathrm{rmsre(X)} = \sqrt{\frac{1}{n}\sum_{i=1}^{n}\left(100\,\frac{X_{\mathrm{meas},i} - X_{\mathrm{sim},i}}{X_{\mathrm{meas},i}}\right)^2} \tag{S2}$$

The quantity X can be either retention times ${}^1t_R$, ${}^2t_R$ or the full widths at half maximum ($FWHM$) ${}^1FWHM$ or ${}^2FWHM$.

Full width at half maximum

The $FWHM$ of a chromatographic peak is estimated by identifying the width of the peak at half its height. Starting from the peak maximum an algorithm searches left and right for the first data points below the half-maximum. Linear interpolation is then used to determine the crossing points and the $FWHM$ is calculated as the difference of these two points. This method provides a robust estimate of peak width, even for asymmetric or non-Gaussian peak shapes, based solely on the experimental signal. In under-sampled peaks, such as those in the first dimension of GC×GC with relatively long modulation periods, a peak may consist of only one or two non-zero points. In such cases, interpolation becomes unreliable or impossible, and the $FWHM$ cannot be robustly estimated. This limitation must be considered when interpreting peak widths from coarse or sparse data.

The peak width τ_R from the simulation is identical with the standard deviation of a gaussian peak. The $FWHM$ of a gaussian peak can be calculated as:

$$FWHM = 2\sqrt{2\ln 2}\,\tau_R \tag{S3}$$

Normalized Euclidean Distance in Two-Dimensional Retention Time Space

To quantify the absolute difference between measured and simulated retention times in GC×GC, a Euclidean distance metric is calculated in the 2D retention time space. Retention times of each dimension are normalized to the maximum. This normalization ensures that both dimensions contribute equally to the distance metric, avoiding bias toward the first dimension, which typically has a much larger scale in GC×GC.

The retention times from both dimensions are concatenated into single vectors for both the measured $\boldsymbol{v}_{\mathrm{meas}}$ and simulated $\boldsymbol{v}_{\mathrm{sim}}$ data.

The Euclidean distance is then computed as:

$$d_e = \|\boldsymbol{v}_{\text{meas}} - \boldsymbol{v}_{\text{sim}}\| \tag{S4}$$

Under normalization, the resulting distance is dimensionless and lies within the interval of 0 to $\sqrt{2}$, representing the geometric deviation between patterns. Table S7 presents the normalized Euclidian distances for the eight measurements with different corrections applied.

Table S9. Normalized Euclidian distance between the 2D retention times of the measurements and the simulations.

Measurement	original	+ t_{MP} corr.	+ t_{shift} corr.
**I-3-3**	1.45	0.90	0.18
**II-5-3**	1.14	0.71	0.12
**III-10-3**	1.40	0.99	0.24
**IV-3-6**	0.88	0.47	0.07
**V-3-4**	0.92	0.48	0.07
**VI-5-4**	1.48	0.98	0.16
**VII-3-3**	1.49	0.88	0.17
**VIII-5-3**	1.26	0.77	0.14

Normalized Cosine Similarity of Retention Time Patterns

Another metric to assess the agreement between simulated and measured retention times in GC×GC is the normalized cosine similarity. The metric compares the retention time vectors (^1t_R, ^2t_R) from simulation and experiment, while accounting for potential scale differences between dimensions.

Retention times in each dimension are independently scaled by the maximum value across simulated and measured data to reduce dimensional imbalance. The retention time vectors are constructed by concatenating the normalized first- and second-dimension retention times. The cosine similarity is defined as:

$$\cos(t_{R,meas}, t_{R,sim}) = \frac{t_{R,meas} \cdot t_{R,sim}}{\left\|t_{R,meas}\right\| \left\|t_{R,sim}\right\|} \tag{S5}$$

This metric yields a value between 0 and 1, where 1 indicates perfect alignment (i.e., simulated and measured retention time patterns differ only by a common scaling factor). Compared to Euclidean distance, cosine similarity emphasizes pattern similarity rather than absolute values, making it robust against systematic shifts or scaling differences between simulations and experiments. This also results in already high values of the cosine similarity for the initial simulations, as the patterns of the 2D chromatogram are similar between measurements and simulations but obvious shifts of the retention times are present. Table S8 presents the cosine similarities for the eight measurements with different corrections applied.

Table S10. Cosine similarity between the 2D retention times of measurements and the simulations using different corrections.

Measurement	original	+ t_{mp} corr.	+ t_{shift} corr.
**I-3-3**	0.9791	0.9927	0.99966
**II-5-3**	0.9816	0.9932	0.99975
**III-10-3**	0.9772	0.9893	0.99934
**IV-3-6**	0.9877	0.9964	0.99992
**V-3-4**	0.9864	0.9864	0.99991
**VI-5-4**	0.9729	0.9884	0.99953
**VII-3-3**	0.9782	0.9930	0.99966
**VIII-5-3**	0.9804	0.9931	0.99971

S.7 Measured and predicted retention times and peak widths

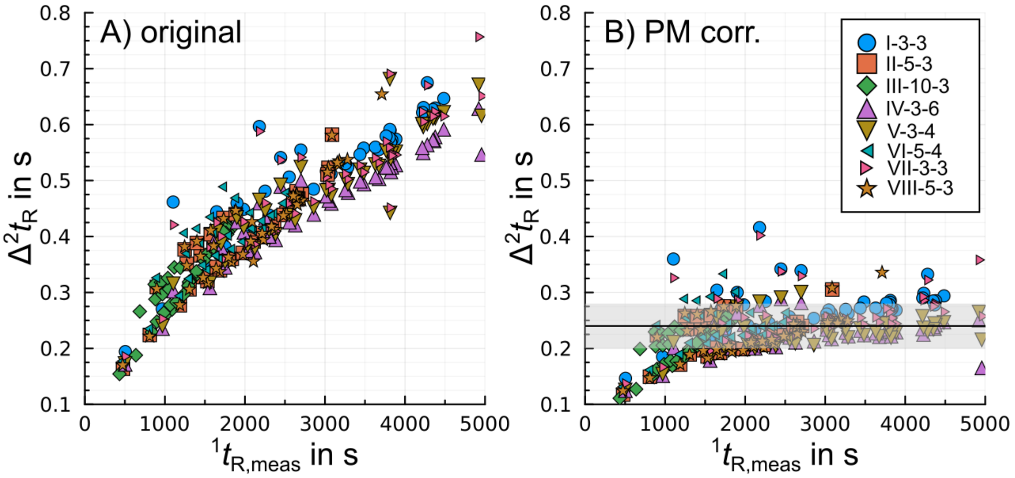

Figure S6. Deviation between measured and predicted 2nd dimension retention time plotted over the measured 1st dimension retention time for A) using the assumed nominal values for modulation and B) for corrected modulation periods.

The measured and predicted values of the first- and second-dimension retention times and peak widths (as FWHM) for the eight measured chromatograms can be found in the excel file '**SuppMat_Data_RT_FWHM.xlsx**'.

S.8 Code examples

Details on how to use the Julia package `GasChromatographySystems.jl` [3] can be found in the supplemental material of [4] and in the package documentation, which will be updated in the future:

https://gaschromatographytoolbox.github.io/GasChromatographySystems.jl/stable/.

Below are selected example scripts illustrating how to define a GC×GC system with thermal modulation and run a simulation. These examples assume that the package and its dependencies have been installed.

Defining temperature and pressure program:

A temperature program ‘`TP`’, for example starting with 40°C, holding for 2 min, heating with 10°C/min to 300°C, holding for 5 min, and a parallel running pressure program ‘`PP`’, for example with inlet pressure of 100 kPa(g) at 40°C and 200 kPa(g) at 300°C can be defined as followed:

```
time_steps, temp_steps = GasChromatographySimulator.conventional_program([40.0, 2.0, 10.0, 300.0, 5.0])
TP = GasChromatographySystems.TemperatureProgram(time_steps, temp_steps)
PP = GasChromatographySystems.PressureProgram(time_steps, [100.0, 100.0, 200.0, 200.0].*1000.0.+101300.0)
```

Function to define a GCxGC system with two thermal modulated points:

```
function GCxGC_TM_8(L1, d1, df1, sp1, TP1, L2, d2, df2, sp2, TP2, LTL, dTL, dfTL,
spTL, TPTL, LM::Array{Float64,1}, dM, dfM, spM, shift, PM, ratioM, HotM, ColdM, TPM,
F, pin, pout; name="GCxGC_TM", opt=GasChromatographySystems.Options(),
optTM=ModuleTMOptions(), optCol=ModuleColumnOptions())
    # graph
    g = SimpleDiGraph(8)
    add_edge!(g, 1, 2) # 1st-D GC
    add_edge!(g, 2, 3) # modulator
    add_edge!(g, 3, 4) # hot/cold 1
    add_edge!(g, 4, 5) # modulator
    add_edge!(g, 5, 6) # hot/cold 2
    add_edge!(g, 6, 7) # 2nd-D GC
    add_edge!(g, 7, 8) # TL

    if pout == 0.0
        pouts = eps(Float64)
    else
        pouts = pout
    end
    # pressurepoints
    pp = Array{GasChromatographySystems.PressurePoint}(undef, nv(g))
    pp[1] = GasChromatographySystems.PressurePoint("p1", pin) # inlet
    for i=2:(nv(g)-1)
        pp[i] = GasChromatographySystems.PressurePoint("p$(i)", NaN)
    end
    pp[end] = GasChromatographySystems.PressurePoint("p$(nv(g))", pouts) # outlet
    # modules
    modules = Array{GasChromatographySystems.AbstractModule}(undef, ne(g))
    modules[1] = GasChromatographySystems.ModuleColumn("GC column 1", L1, d1*1e-3,
df1*1e-6, sp1, TP1, F/60e6, optCol)
    modules[2] = GasChromatographySystems.ModuleColumn("mod in", LM[1], dM*1e-3,
dfM*1e-6, spM, TPM, optCol)
    modules[3] = GasChromatographySystems.ModuleTM("TM1", LM[2], dM*1e-3, dfM*1e-6,
spM, TPM, shift, PM, ratioM, HotM, ColdM, NaN, optTM)
    modules[4] = GasChromatographySystems.ModuleColumn("mod loop", LM[3], dM*1e-3,
dfM*1e-6, spM, TPM, optCol)
    modules[5] = GasChromatographySystems.ModuleTM("TM2", LM[4], dM*1e-3, dfM*1e-6,
spM, TPM, shift, PM, ratioM, HotM, ColdM, NaN, optTM)
    modules[6] = GasChromatographySystems.ModuleColumn("GC column 2", L2, d2*1e-3,
df2*1e-6, sp2, TP2, NaN, optCol)
    modules[7] = GasChromatographySystems.ModuleColumn("TL", LTL, dTL*1e-3, dfTL*1e-
6, spTL, TPTL, NaN, optCol)
    # system
    sys =
GasChromatographySystems.update_system(GasChromatographySystems.System(name, g, pp,
modules, opt))
    return sys
end
```

Parameters '`L1, d1, df1, sp1, TP1`' describe the parameters of the first-dimension column (length, diameter, film thickness, stationary phase, and temperature program), parameters '`L2, d2, df2, sp2, TP2`' define the second-dimension column after the modulator, parameters '`LTL, dTL, dfTL, spTL, TPTL`' define the transfer line to the detector, and '`LM::Array{Float64,1}, dM, dfM, spM, shift, PM, ratioM, HotM, ColdM, TPM`' describe the modulator with four values for '`LM`' (length before the first modulation point, length of the first modulation point, length between modulation points, and length of second modulation point) and '`ratioM`' is the ration between time of active cold-jet to time of active hot-jet. The value for '`F`' (column flow) respectively '`pin`' (inlet pressure) is either 'NaN', a number or a program. If '`pin`' is 'NaN' the flow '`F`' must be defined or the other way around. '`pout`' is the outlet pressure.

With the usage of the function the system '`sys`' is defined as followed:

```
sys = GCxGC_TM_8( 29.74, 0.25, 0.25, "ZB1ms", TP,
                0.53, 0.1, 0.1, "Stabilwax", TP,
                0.24, 0.1, 0.1, "Stabilwax", 300.0,
                [0.3, 0.05, 0.9, 0.05], 0.1, 0.1, "Stabilwax",
                0.13, 3.00236, (3.000236-0.35)/0.35, 25.0, -80.0, TP,
                NaN, PP, 0.0)
```

To calculate the (squared) pressures '`p2fun`' at the connection points of the capillaries (vertices):

```
p2fun = GasChromatographySystems.build_pressure_squared_functions(sys,
GasChromatographySystems.solve_balance(sys))
```

The list of parameters '`par`' of each segment is created by:

```
par = GasChromatographySystems.graph_to_parameters(sys, p2fun, db, solute_values)
```

At this point a database of retention parameters '`db`' and a list of the names of solutes '`solute_values`', which should be simulated are needed. On the GitHub repository of GasChromatographySystems.jl the retention data used for this publication can be loaded with:

```
db =
DataFrame(urldownload("https://github.com/GasChromatographyToolbox/GasChromatography
Systems.jl/blob/main/data/Database_GCxGC-TM.csv"))
```

The simulation itself is started with:

```
sim = GasChromatographySystems.simulate_along_paths(sys, p2fun,
GasChromatographySystems.all_paths(sys.g, 1)[2], par)
```

The function '`all_paths`' defines the order of the '`par`' entries for the simulated path, whereby '`1`' indicates, that only one path is possible.

From the simulation result a peak list with two-dimensional retention data and peak widths can be extracted using the modulation period:

```
pl_GCxGC = GasChromatographySystems.peaklist_GCxGC(sim[2][1][end], 3.000236)
```

These code snippets provide a reproducible starting point for simulating thermally modulated GC×GC separations using the modular graph-based approach described in the main manuscript.

S.9 Pluto notebook

A Pluto notebooks with a demo version for the simulation of GC×GC with thermal modulation can be found in the subfolder `notebooks/ GCxGC-TM_Paper` in the Github repository of the GasChromatographySystems.jl package.

To use this notebook, first the Julia Programming Language (https://julialang.org/downloads/) must be installed. Next, the package Pluto.jl has to be added. After starting Julia in the terminal type `]` to enter the package manager and then type `add Pluto` and the package will be downloaded and installed. This has to be done only once. To use the package, leave the package manager by pressing the Backspace key and then enter `using Pluto; Pluto.run()`. The following interface will be opened in the browser:

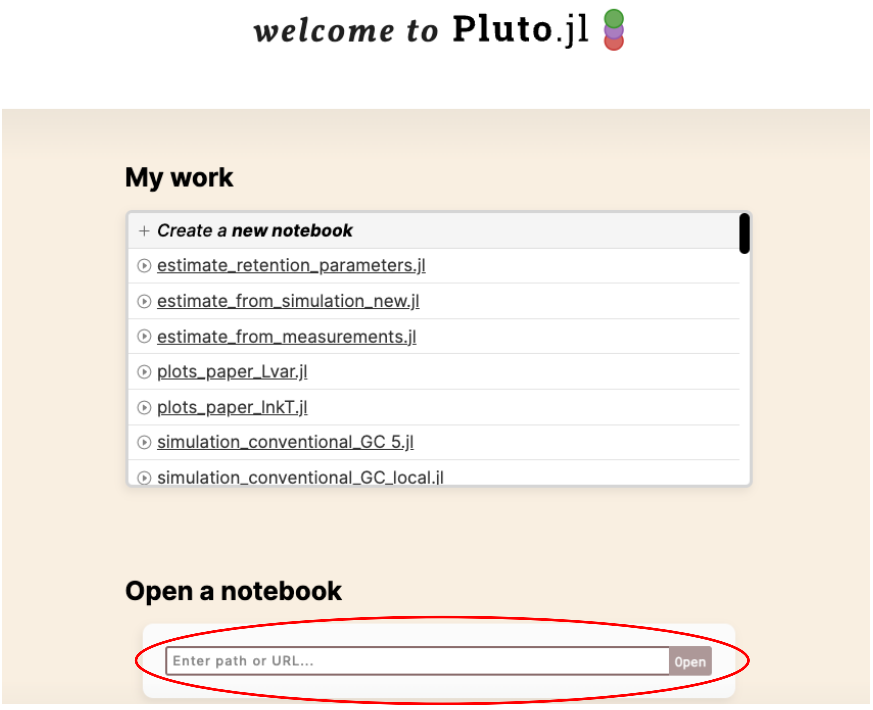

Under “Open a notebook” the following URL can be entered:

https://github.com/GasChromatographyToolbox/GasChromatographySystems.jl/blob/main/notebooks/GCxGC-TM_Paper/GCxGC_TM_Demo.jl

S.10 References

[1] Jan Leppert, GasChromatographyToolbox/RetentionParameterEstimator.jl: v0.2.0. 2024, DOI: 10.5281/ZENODO.14176463.

[2] Leppert, J., Brehmer, T., Wüst, M., Boeker, P., Estimation of retention parameters from temperature programmed gas chromatography. *Journal of Chromatography A* 2023, 1699, 464008.

[3] Jan Leppert, GasChromatographyToolbox/GasChromatographySystems.jl: v0.2.6. 2025, DOI: 10.5281/ZENODO.16576985.

[4] Leppert, J., Brehmer, T., Boeker, P., Wüst, M., Generalized flow calculation of the gas flow in a network of capillaries used in gas chromatography. *J of Separation Science* 2024, 47, 2400419.
